# Effects of PD-1 Signaling on Immunometabolic Reprogramming

**DOI:** 10.20900/immunometab20220007

**Published:** 2022-03-10

**Authors:** Vassiliki A. Boussiotis, Nikolaos Patsoukis

**Affiliations:** 1 Division of Hematology-Oncology, Beth Israel Deaconess Medical Center, Harvard Medical School, Boston, MA 02215, USA; 2 Department of Medicine, Beth Israel Deaconess Medical Center, Harvard Medical School, Boston, MA 02215, USA

**Keywords:** PD-1, metabolic reprogramming, T cells, immunometabolism, adaptive and innate immunity, T cell exhaustion

## Abstract

Programmed Death-1 (PD-1; CD279) is an inhibitory receptor induced in several activated immune cells and, after engagement with its ligands PD-L1 and PD-L2, serves as a key mediator of peripheral tolerance. However, PD-1 signaling also has detrimental effects on T cell function by posing breaks on antitumor and antiviral immunity. PD-1 blocking immunotherapy either alone or in combination with other therapeutic modalities has shown great promise in cancer treatment. However, it is unclear why only a small fraction of patients responds to this type of therapy. For this reason, efforts to better understand the mechanisms of PD-1 function have recently been intensified, with the goal to reveal new strategies to overcome current limitations. The signaling pathways that are inhibited by PD-1 impact key regulators of metabolism. Here, we provide an overview of the current knowledge about the effects of PD-1 on metabolic reprogramming of immune cells and their consequences on systemic metabolism.

## INTRODUCTION

PD-1 and its ligands are components of a central inhibitory pathway upregulated upon activation in several cell types of the adaptive and innate immune system. Its main function is to prevent autoimmunity while keeping immune responses efficient and balanced [1–4]. However, PD-1 signaling prevents antitumor immunity by interacting with its ligands expressed on cancer [5] and antigen presenting cells (APC) [6,7] within the tumor microenvironment (TME). These findings led to the development of blocking therapeutic antibodies against PD-1 and its ligands that revolutionized cancer immunotherapy [8–10]. The limited success of this therapeutic approach has recently sparked extensive efforts to better understand the mechanisms of PD-1 signaling and function with the goal to improve the therapeutic success.

Besides the recent advancements in understanding the molecular mechanisms of its function (extensively reviewed in [11] and [12]), efforts have focused in understanding metabolic implications of PD-1 signaling. Extensive studies have documented that PD-1 is a central regulator of a metabolically dysfunctional state termed “exhaustion” which occurs in T cells under conditions of persistent activation as in the case of cancer and chronic viral infections [13]. Nevertheless, genetic deletion of PD-1 not only was insufficient to rescue cells from this metabolic dysfunction, but actually promoted accumulation of terminally differentiated exhausted CD8^+^ T cells [14]. This finding suggested that overcoming exhaustion cannot be achieved solely by targeting PD-1, since a plethora of other inhibitory receptors, such as LAG-3, CD244 (also known as 2B4), CD160, CTLA-4 and TIM-3 [15–18] as well as factors in the TME beyond checkpoint inhibitors can contribute to this dysfunctional metabolic state [19]. By altering TCR signaling, PD-1 results in alterations of immunometabolic reprogramming thereby imprinting transcriptional, metabolic and epigenetic implications on the differentiation and function of immune cells. Here, we provide a brief overview of the current knowledge regarding the effects of PD-1 on the metabolic features of immune cells.

## PD-1-MEDIATED METABOLIC REPROGRAMMING OF IMMUNE CELLS

Signaling and metabolism are two tightly linked entities. Signaling can cause metabolic changes, whereas, conversely, metabolic changes can alter the expression of signaling molecules through epigenetic and transcriptional control. PD-1 is a surface inhibitory receptor upregulated on activated immune cells and acts at the forefront of immune signaling.

### T Cells

#### PD-1 impact on T cell signaling pathways and link with metabolism

**Figure 1.**
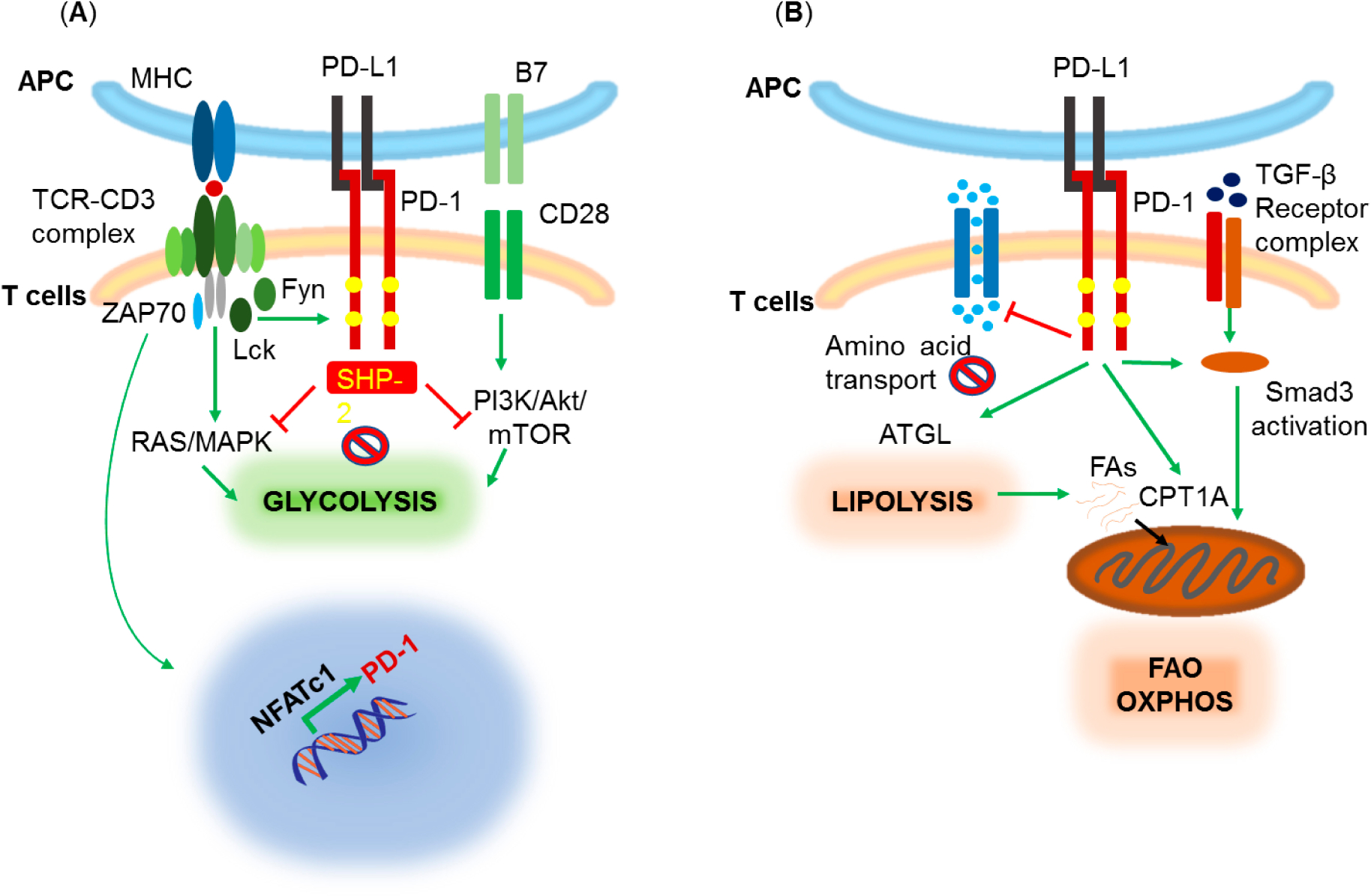
Effect of PD-1 on T cell-mediated signaling pathways and metabolism. (**A**) TCR stimulation induces NFATc1-mediated PD-1 upregulation. PD-1 is phosphorylated by TCR-proximal Src-family kinases Lck and Fyn and recruits SHP-2 phosphatase which downregulates TCR- and CD28-mediated signals thereby preventing induction of glycolysis by these pathways. (**B**) Besides, glycolysis, PD-1 inhibits amino acid transport and metabolism and promotes lipolysis and FAO by inducing expression of ATGL and CPT1A, respectively. PD-1 also enhances the TGF-β signaling through Smad3 activation, a pathway known to promote FAO, OXPHOS, and Treg differentiation.

PD-1 has been mostly studied in T cells and upregulation of PD-1 expression is tightly correlated with T cell activation. TCR signaling induces PD-1 expression through the transcriptional activator NFATc1 [20] (Figure 1). It has been shown that artificial increase of basal TCR signaling by expressing the semi-active mutant form of ZAP70 W131A, which disrupts normal ZAP70 autoinhibition, triggered a feedback increase in inhibitory receptor expression including PD-1 and resulted in T cell unresponsiveness, partially reversible by PD-1 blockade [21]. In addition, PD-1 upregulation in T cells can be induced by the Notch signaling pathway [22]. PD-1 ligation by its ligands PD-L1 or PD-L2, concomitantly with TCR signaling, results in Src-family kinase (Lck and/or Fyn)-mediated tyrosine phosphorylation of the PD-1 cytoplasmic tail at the immunoreceptor tyrosine inhibitory motif (ITIM) and at the immunoreceptor tyrosine switch motif (ITSM) leading to the recruitment of phosphatase SHP-2 which then downregulates signaling pathways associated with the T cell receptor (TCR) and the CD28 costimulatory receptor [23–36] (Figure 1A). Two main target pathways include Ras/MAPK and PI3K/Akt/mTOR, which are key regulators of T cell metabolism, primarily integrating signals from the TCR, costimulatory and cytokine receptors to promote a glycolytic phenotype [37,38] (Figure 1A). PD-1 counteracts these pathways, and keeps T cells “on a diet” as it decreases glycolysis, amino acid transport and metabolism [27,39]. Concomitantly, PD-1 signaling promotes fatty acid oxidation (FAO) of endogenous lipids by increasing expression of carnitine palmitoyltransferase 1A (CPT1A), and induces lipolysis by increasing expression of adipose triglyceride lipase (ATGL) [39] (Figure 1B).

Notably, PD-1 engagement can also increase polyunsaturated fatty acids (PUFA) intracellularly and in culture supernatants [39]. PUFAs can inhibit T cell proliferation and interleukin-2 (IL-2) production in vitro and in vivo [40,41]. Due to the presence of two or more carbon–carbon double bonds PUFA are sensitive to oxidation by reactive oxygen species (ROS). Notably, uptake of oxidized lipids by the scavenger receptor CD36 can promote lipid peroxidation and dysfunction of CD8^+^ T cells in tumors [42]. Thus, PD-1-mediated increase of PUFA may be an important mechanism contributing to T cell dysfunction by increasing oxidized lipids in the TME.

Little is known about the effects of PD-1 on the redox metabolism of T cells. Metabolomics analysis of T cells subjected to PD-1 ligation showed decreased levels of reduced glutathione GSH and higher levels of cysteine-GSH disulfide indicative of a more oxidative environment in T cells receiving PD-1 signals [39]. A recent study found that alloreactive T cells from a mouse graft versus host disease (GVHD) model had increased expression of PD-1 and FAO-derived ROS, which made the cells more susceptible to F1F0-ATP synthase complex inhibitors [43]. These effects were reverted either by antioxidants or by PD-1 blockade, resulting in decreased cellular ROS, making the cells resistant to F1F0-ATP inhibition [43]. In contrast, other investigators reported that PD-1 blockade increased cellular ROS and mitochondrial mass together with proliferation and activation of CD8^+^ T cells in the tumor microenvironment [44].

Another pathway involved in T cell metabolism that is targeted by PD-1, particularly in regulatory T cells (Treg), is the TGF-β pathway. TGF-β counteracts the PI3K pathway and promotes FAO to fuel mitochondrial oxidative phosphorylation (OXPHOS) [45]. PD-1 favors this metabolic phenotype [39]. In parallel, PD-1 reduces the threshold of TGF-β-mediated Treg development [46] while enhancing SMAD3 transactivation [31] (Figure 1B). TGF-β attenuates anti-tumor responses mediated by PD-L1 blocking immunotherapy in patients and murine tumor models by promoting a T cell exhaustion phenotype. Conversely, simultaneous blockade of PD-1 and TGF-β-mediated signaling, improved responses in patients with various cancers [47]. Notably, recent preclinical studies employing bifunctional fusion proteins blocking the PD-1 pathway in conjunction with TGF-β showed significant enhancement of antitumor activity [48,49] and such outcomes were also confirmed in patients [50,51].

Another mechanism promoting PD-1-mediated T cell dysfunction in chronic infections, such as HIV in humans and LCMV in mice, is the upregulation of the basic leucine zipper ATF-like transcription factor (BATF) [52]. Interestingly, BATF was found to couple triglyceride (TG) metabolism with Treg suppressive function for controlling allergic airway inflammation and IgE responses [53]. However, the role of BATF in T cell exhaustion remains controversial, as recent studies supported a beneficial role of BATF on T cell responses in chronic infection [54] and cancer [55].

#### PD-1-mediated metabolic effects on T cell memory

Besides suppressing activation and IL-2 production by T effector cells, thereby inducing a state of anergy [56], PD-1 also affects T cell memory differentiation as T cells from PD-1 knockout mice are skewed towards an effector (T_EM_) over a central (T_CM_) memory phenotype [57]. By using single-cell RNA-sequencing, a recent study identified two previously unrecognized distinct stem-like CD8^+^ memory T cell subsets with distinct fate commitments in humans, one lacking PD-1 and T cell immunoreceptor with Ig and ITIM domains (TIGIT) committed to a functional lineage, and a second expressing PD-1 and TIGIT committed to a dysfunctional, exhausted-like lineage [58]. However, it was previously observed that complete lack of PD-1 signaling was not beneficial for long-term memory differentiation, but resulted in the generation of exhausted, short lived, terminally differentiated CD8^+^ T cells [14]. Notably, recent reports documented that PD-1 plays a critical role in efficient memory differentiation by preserving a key memory CD8^+^ T cell precursor pool during initial activation, and supporting its persistence after antigen clearance [59,60]. Collectively, PD-1 acts as a key metabolic regulator of T cell memory, a critical function that should be taken into consideration when PD-1 blockade therapy is designed.

#### The role of PD-1 on Treg function and metabolism

As mentioned above, PD-1 promotes Treg differentiation by lowering the threshold of TGF-β signaling [46]. Upregulation of PD-1 and other inhibitory receptors on tumor-infiltrating Treg cells had been correlated with their enhanced suppressive function [61]. A surprising recent finding was that PD-1 blockade, besides inducing recovery of dysfunctional PD-1^+^ T cells, enhances the immunosuppressive function of PD-1^+^ Treg thereby promoting cancer hyperprogression [62,63]. These observations suggest a negative impact of PD-1 on Treg suppression activity and indicate that the balance of PD-1 expression between T effector and Treg cells may be a predictive biomarker of the clinical efficacy of PD-1 blocking immunotherapy [63]. Notably, in the tumor microenvironment, Treg cells but not FoxP3^−^CD4^+^ or CD8^+^ T cells have the ability to utilize lactic acid produced by highly glycolytic cancer cells, through MCT1-mediated import, conferring metabolic advantage and survival. Lactic acid-driven metabolism and activation of Treg in the tumor microenvironment results in preferential upregulation of PD-1 in Treg cells leading to imbalanced PD-1 expression between T effector and Treg and impairment of PD-1 blocking immunotherapy [64].

**Figure 2.**
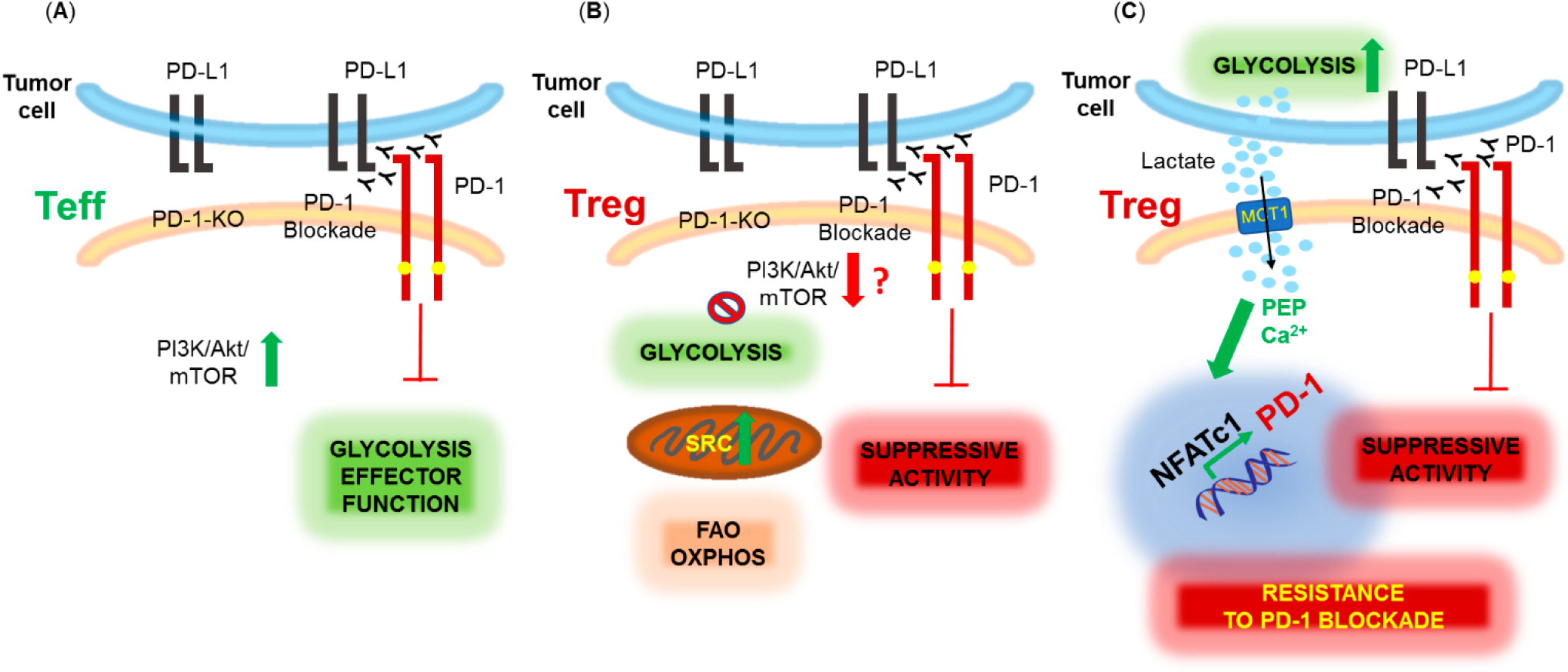
Metabolic effects of PD-1 ablation or blockade in effector (Teff) and regulatory (Treg) T cells. (**A**) PD-1 inhibits the effector function of Teff cells which is restored by PD-1 genetic ablation or blockade, promoting activation of the PI3K/Akt/mTOR pathway and glycolysis. (**B**) PD-1 blockade or ablation enhances the suppressive function of Treg, possibly, by downregulating the PI3K/Akt/mTOR pathway and glycolysis and promoting mitochondrial activity and spare respiratory capacity (SRC). (**C**) Highly glycolytic tumors produce high levels of lactic acid (lactate) in the TME. Lactate is uptaken mainly by Treg cells expressing monocarboxylate transporter 1 (MCT1) and converted into phosphoenol pyruvate (PEP) leading to increased calcium (Ca^2+^) in the cytoplasm, NFATc1 translocation into the nucleus and upregulation of PD-1 expression. PD-1 blockade under conditions leading to this metabolic adaptation in Treg cells enhances Treg suppressive function resulting in resistance to PD-1 blockade therapy.

Specific effects of PD-1 on Treg function and metabolism had not been directly assessed until recently, when mice with Treg-specific PD-1 deletion were generated [65]. In agreement with previous observations [62,63] the study found that lack of PD-1 expression in Treg cells increased their suppressive function and demonstrated the in vivo significance of this effect by the improved outcomes in mouse models of autoimmunity, such as experimental autoimmune encephalomyelitis (EAE) and nonobese diabetes (NOD) selectively lacking PD-1 in Treg cells [65]. Since PD-1 engagement inhibits the PI3K/Akt pathway on effector T cells [26,31,46], and promotes lipid oxidation [39], a preferred metabolic pathway in Treg cells [66], it would be anticipated that genetic ablation of PD-1 in Treg would enhance activation of the PI3K/Akt pathway and glycolysis. In contrast, PD-1-deficient Treg cells were found to have decreased glycolytic activity due to reduced PI3K/Akt/mTOR signaling [65]. This finding, although surprising, could explain the enhanced suppressive activity of PD-1-deficient Treg as inhibition of the PI3K/Akt/mTOR pathway is necessary for the suppressive function of Treg cells [67,68]. In addition, activated PD-1-deficient Treg cells had increased mitochondrial spare respiratory and maximal respiratory capacity and displayed a bioenergetic state characterized by enhanced mitochondrial function but reduced glycolysis [65]. The mechanisms of these effects of PD-1 ablation in Treg remain elusive. Collectively, the role of PD-1 is to inhibit the function of T effector cells and Treg cells, and genetic ablation or blockade of PD-1 can reverse this outcome, albeit by inducing opposite metabolic signatures in these two cell types (Figure 2).

### Innate Immune Cells

Although PD-1 has been mostly studied in T cells, it is increasingly being appreciated that is also upregulated in other immune cell types including innate immune cells such as macrophages [69–71], tumor-associated macrophages (TAMs) [72], monocytes [73,74] myeloid progenitors and myeloid-derived suppressor cells (MDSC) [75] and innate lymphoid cells (ILCs) such as natural killer (NK) cells [76], and regulates their differentiation and function as previously extensively reviewed [11,77–79].

#### Myeloid cells

**Figure 3.**
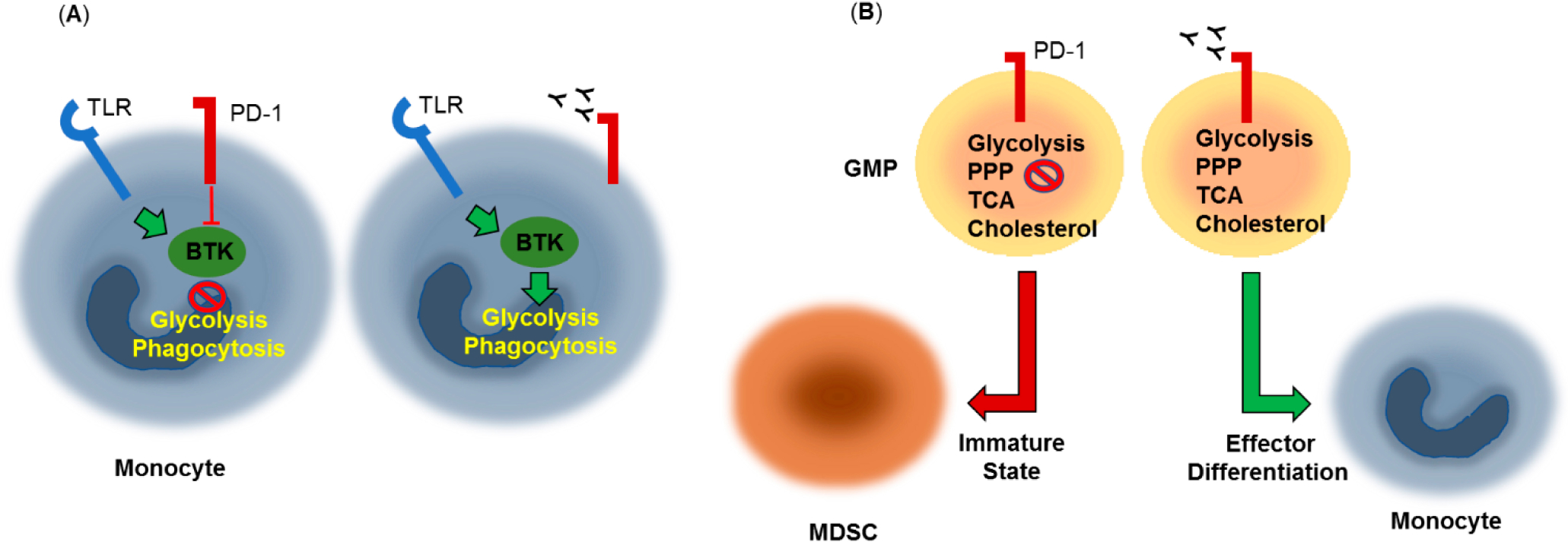
Metabolic effects of PD-1 in myeloid cells. (**A**) PD-1 triggering on monocytes results in impaired Bruton’s tyrosine kinase (BTK) signaling, glycolysis and phagocytosis. PD-1 pathway blockade reverts this metabolic dysfunction. TLR: Toll-Like Receptor. (**B**) Granulocyte/macrophage progenitors (GMPs), which accumulate during cancer-driven emergency myelopoiesis express PD-1, which compromised growth factor-mediated signaling and metabolic activity, preserving an immature state and giving rise to myeloid-derived suppressor cells (MDSCs). PD-1 ablation or blockade prevents MDSC accumulation, and promotes effector differentiation. These effects are correlated with increased metabolic activity of glycolysis, pentose phosphate pathway (PPP), and TCA cycle and elevated cholesterol, a key enhancer of antigen-presenting function.

Limited information is currently available regarding the effects of PD-1 on the signaling and metabolic reprogramming of myeloid cells. A recent study examining immune dysfunctions in chronic lymphocytic leukemia (CLL) found that PD-1 triggering on monocytes resulted in impaired glycolysis, phagocytosis and Bruton’s tyrosine kinase (BTK) signaling and that blocking the PD-1 pathway reverted the metabolic dysfunction [74] (Figure 3A).

It was recently shown that granulocyte/macrophage progenitors (GMPs), which accumulate during cancer-driven emergency myelopoiesis and give rise to myeloid-derived suppressor cells (MDSCs), express PD-1 and PD-L1 [75]. Myeloid-specific ablation of PD-1 in tumor-bearing mice, prevented MDSC accumulation, while increasing systemic output of effector myeloid cells and T effector memory cells with improved antitumor function. These outcomes were correlated with metabolic alterations in PD-1-deficient myeloid progenitors in response to factors that drive emergency myelopoiesis characterized by increased intermediates of glycolysis, pentose phosphate pathway, and TCA cycle but, most prominently, elevated cholesterol, a key mediator of inflammatory differentiation of macrophages and DC favoring antigen-presenting function [80] (Figure 3B). Thus, skewing myeloid differentiation towards effector and inflammatory antigen presenting cells rather than immunosuppressive MDSC might be a key mechanism of antitumor immunity mediated by PD-1 blockade.

#### NK cells

NK cells form an important component of the innate lymphoid system [81,82]. Despite their lack of antigen-specific receptors they have significant antiviral and antitumor activity through antigen-non-specific mechanisms and, similarly to T cells, their effector function relies on activation of the PI3K/Akt/mTOR pathway and glycolysis [83,84]. Upon activation, NK cells, and other ILCs, upregulate PD-1 [76]. However little is known about the role of PD-1 on the metabolic properties on NK cells. Similarly to T cells, PD-1 has an inhibitory role in NK cell function. In the context of several human cancers and mouse tumor models, PD-1 expression in NK cells has been correlated with a dysfunctional, exhausted phenotype, amenable to reversion by PD-1 blockade [85–88]. However, recent studies provided evidence for a different role of NK-expressed PD-1 in controlling host resistance to viral infection. Endogenous glucocorticoids are essential steroid hormones produced after activation of the hypothalamic-pituitary-adrenal (HPA) neuroendocrine axis in response to infection. They bind to cytosolic glucocorticoid receptors (GR) thereby mediating signals that lead to resolution of inflammation and maintenance of immune homeostasis. A recent study in a mouse model provided evidence that GR-mediated signaling in response to CMV infection induced tissue-specific PD-1 expression on NK cells. GR-mediated PD-1 expression diminished the production of IFN-γ by in NK cells and prevented lethal immunopathology without compromising viral clearance [89]. A study in human tumor samples showed that glucocorticoids present in the TME in combination with inflammatory cytokines IL-12, IL-15, and IL-18 increased PD-1 expression on NK cells and induced an immunosuppressive milieu [90]. These studies revealed a new mechanism by which PD-1 expression in innate immune cells is controlled by a neuroendocrine-immune axis, thereby regulating immune homeostasis but also compromising anti-tumor immunity.

These evolving observations reveal that PD-1 has a previously unappreciated role in the innate immune system by affecting several properties and functions of innate immune cells including metabolic and differentiation programs.

## PD-1 EFFECTS ON SYSTEMIC METABOLISM

Releasing the PD-1 break on immune cells not only affects the targeted cells but can also have broader systemic impact on the metabolic regulation of the whole organism. For example, one of the first observations regarding the role of PD-1 in preventing autoimmunity was that PD-1 loss or blockade promoted the development of type 1 diabetes in mice [91,92]. Other early studies showed that impairment of the PD-1 pathway enhanced and exacerbated the development of atherosclerotic lesion in proatherogenic mice [93,94]. Recently, similar effects were observed in patients treated with PD-1 blocking checkpoint immunotherapy [95].

The systemic consequences of the metabolic alterations related to ablation of PD-1 signaling were extensively examined by analyzing water-soluble metabolites in the serum of PD-1 deficient mice, which develop a spontaneous activated phenotype [96]. Differences were observed in several metabolites related to energy-generating pathways, such as the TCA cycle, glucose and amino-acid metabolism. The most significant change affected the abundance of the aromatic amino acids tryptophan, tyrosine and phenylalanine, which were much lower in the PD-1 deficient mice compared to control, by 2 months of age. This was due to the increased intracellular transport and utilization of these amino acids by activated T cells and resulted in depletion of tyrosine and tryptophan from the brain, where these are essential for synthesis of the neurotransmitters dopamine (DA) and serotonin (5-hydroxytryptamine (5-HT)). The consequence was a substantial deficiency in both these neurotransmitters in the brain, resulting in behavioral changes associated with anxiety behavior and exacerbated fear responses [96].

Systemic metabolic adaptations to PD-1 blockade immunotherapy were also detected in human patients. Comprehensive analysis of serum metabolites in patients with advanced melanoma and renal cell carcinoma treated with the anti-PD-1 antibody nivolumab, identified increase of kynurenine/tryptophan ratio. This finding, indicative of increased activity of indoleamine 2,3-dioxygenase (IDO), was associated with worse overall survival, suggesting that increase of IDO activity after PD-1 checkpoint immunotherapy might be a mechanism of systemic adaptive immune resistance [97]. Conversely, as determined by a different study using samples from patients treated with checkpoint immunotherapy, elevated metabolites derived from the microbiome (hippuric acid), fatty acid oxidation (butyrylcarnitine) and redox metabolism (cystine and glutathione disulfide), provided high response probability. In the same patients, systemic increase of CD4^+^ and CD8^+^ T cells combined with elevated expression of PGC-1 and ROS were also correlated with improved responses to nivolumab [98].

Collectively, these findings suggest that interfering with PD-1 function on immune cells may have consequences that extend beyond the immune system. Moreover, metabolic monitoring might have a predictive value of therapeutic response and might assist in patient stratification for combining metabolic interventions with PD-1 blockade immunotherapy. However, much remains to be learned regarding the effects of PD-1 signaling or its blockade on systemic metabolic adaptation in the context of cancer. Based on the very limited available reports in the literature [96–98] PD-1 blockade resulted in distinct metabolic alterations in the context of different tumors. For example, although activated T cells appeared to have a dominant role in altering systemic metabolic responses in the context of a murine tumor model [96], in human patients treated with checkpoint immunotherapy, metabolic alterations of cancer cells dominated the systemic metabolic outcome [97]. Additional work in human patients and mouse tumor models is required to dissect the determinants of systemic metabolic adaptations to checkpoint immunotherapy and guide rational approaches of systemic metabolic interventions to enhance the therapeutic outcome of checkpoint immunotherapy.

## CONCLUSIONS

It is increasingly being appreciated that besides T cells, PD-1 engagement or blockade have a major impact on metabolic reprogramming of multiple immune cell types and these effects can extend systemically. Although signaling- and metabolism-targeting effects of PD-1 engagement and blockade have been extensively studied in T cells, evolving studies provide evidence for the important implications of PD-1 signaling in several cell types of the innate immune system. Besides metabolomics and bioenergetics on sorted cell populations, novel methodologies such as SCENITH [99] will allow a more accurate metabolic characterization and understanding of PD-1 effects on rare immune populations. It is now apparent that targeting the PD-1 pathway alone is not sufficient to overcome the complex dysfunctional state of various immune cell populations in the tumor microenvironment. Harnessing metabolism combined with interventions to checkpoint pathways holds a great promise against this challenge.

## References

[1] Nishimura H, Nose M, Hiai H, Minato N, Honjo T. Development of lupus-like autoimmune diseases by disruption of the PD-1 gene encoding an ITIM motif-carrying immunoreceptor. Immunity. 1999;11(2):141–51. 10485649 10.1016/s1074-7613(00)80089-8

[2] Nishimura H, Okazaki T, Tanaka Y, Nakatani K, Hara M, Matsumori A, Autoimmune dilated cardiomyopathy in PD-1 receptor-deficient mice. Science. 2001;291(5502):319–22. 11209085 10.1126/science.291.5502.319

[3] Martin-Orozco N, Wang YH, Yagita H, Dong C. Cutting Edge: Programmed death (PD) ligand-1/PD-1 interaction is required for CD8+ T cell tolerance to tissue antigens. J Immunol. 2006;177(12):8291–5. 17142723 10.4049/jimmunol.177.12.8291

[4] Keir ME, Butte MJ, Freeman GJ, Sharpe AH. PD-1 and its ligands in tolerance and immunity. Annu Rev Immunol. 2008;26:677–704. 18173375 10.1146/annurev.immunol.26.021607.090331 PMC10637733

[5] Latchman Y, Wood CR, Chernova T, Chaudhary D, Borde M, Chernova I, PD-L2 is a second ligand for PD-1 and inhibits T cell activation. Nat Immunol. 2001;2(3):261–8. 11224527 10.1038/85330

[6] Dong H, Strome SE, Salomao DR, Tamura H, Hirano F, Flies DB, Tumor-associated B7-H1 promotes T-cell apoptosis: a potential mechanism of immune evasion. Nat Med. 2002;8(8):793–800. 12091876 10.1038/nm730

[7] Curiel TJ, Wei S, Dong H, Alvarez X, Cheng P, Mottram P, Blockade of B7-H1 improves myeloid dendritic cell-mediated antitumor immunity. Nat Med. 2003;9(5):562–7. 12704383 10.1038/nm863

[8] Brahmer JR, Tykodi SS, Chow LQ, Hwu WJ, Topalian SL, Hwu P, Safety and activity of anti-PD-L1 antibody in patients with advanced cancer. N Engl J Med. 2012;366(26):2455–65. 22658128 10.1056/NEJMoa1200694 PMC3563263

[9] Garon EB, Rizvi NA, Hui R, Leighl N, Balmanoukian AS, Eder JP, Pembrolizumab for the treatment of non-small-cell lung cancer. N Engl J Med. 2015;372(21):2018–28. 25891174 10.1056/NEJMoa1501824

[10] Ansell SM, Lesokhin AM, Borrello I, Halwani A, Scott EC, Gutierrez M, PD-1 blockade with nivolumab in relapsed or refractory Hodgkin’s lymphoma. N Engl J Med. 2015;372(4):311–9. 25482239 10.1056/NEJMoa1411087 PMC4348009

[11] Patsoukis N, Wang Q, Strauss L, Boussiotis VA. Revisiting the PD-1 pathway. Sci Adv. 2020;6(38):eabd2712. 32948597 10.1126/sciadv.abd2712 PMC7500922

[12] Wang Q, Bardhan K, Boussiotis VA, Patsoukis N. The PD-1 interactome. Adv Biol. 2021;5(9):e2100758. 10.1002/adbi.202100758 PMC10754315 34170628

[13] Bengsch B, Johnson AL, Kurachi M, Odorizzi PM, Pauken KE, Attanasio J, Bioenergetic insufficiencies due to metabolic alterations regulated by the inhibitory receptor PD-1 are an early driver of CD8(+) T cell exhaustion. Immunity. 2016;45(2):358–73. 27496729 10.1016/j.immuni.2016.07.008 PMC4988919

[14] Odorizzi PM, Pauken KE, Paley MA, Sharpe A, Wherry EJ. Genetic absence of PD-1 promotes accumulation of terminally differentiated exhausted CD8^+^ T cells. J Exp Med. 2015;212(7):1125–37. 26034050 10.1084/jem.20142237 PMC4493417

[15] Wherry EJ, Ha SJ, Kaech SM, Haining WN, Sarkar S, Kalia V, Molecular signature of CD8^+^ T cell exhaustion during chronic viral infection. Immunity. 2007;27(4):670–84. 17950003 10.1016/j.immuni.2007.09.006

[16] Blackburn SD, Shin H, Haining WN, Zou T, Workman CJ, Polley A, Coregulation of CD8^+^ T cell exhaustion by multiple inhibitory receptors during chronic viral infection. Nat Immunol. 2009;10(1):29–37. 19043418 10.1038/ni.1679 PMC2605166

[17] Fourcade J, Sun Z, Benallaoua M, Guillaume P, Luescher IF, Sander C, Upregulation of Tim-3 and PD-1 expression is associated with tumor antigen-specific CD8^+^ T cell dysfunction in melanoma patients. J Exp Med. 2010;207(10):2175–86. 20819923 10.1084/jem.20100637 PMC2947081

[18] Zhou Q, Munger ME, Veenstra RG, Weigel BJ, Hirashima M, Munn DH, Coexpression of Tim-3 and PD-1 identifies a CD8^+^ T-cell exhaustion phenotype in mice with disseminated acute myelogenous leukemia. Blood. 2011;117(17):4501–10. 21385853 10.1182/blood-2010-10-310425 PMC3099570

[19] Kubli SP, Berger T, Araujo DV, Siu LL, Mak TW. Beyond immune checkpoint blockade: emerging immunological strategies. Nat Rev Drug Discov. 2021;20(12):899–919. 33686237 10.1038/s41573-021-00155-y

[20] Oestreich KJ, Yoon H, Ahmed R, Boss JM. NFATc1 regulates PD-1 expression upon T cell activation. J Immunol. 2008;181(7):4832–9. 18802087 10.4049/jimmunol.181.7.4832 PMC2645436

[21] Hsu LY, Cheng DA, Chen Y, Liang HE, Weiss A. Destabilizing the autoinhibitory conformation of Zap70 induces up-regulation of inhibitory receptors and T cell unresponsiveness. J Exp Med. 2017;214(3):833–49. 28159798 10.1084/jem.20161575 PMC5339679

[22] Mathieu M, Cotta-Grand N, Daudelin JF, Thebault P, Labrecque N. Notch signaling regulates PD-1 expression during CD8(+) T-cell activation. Immunol Cell Biol. 2013;91(1):82–8. 23070399 10.1038/icb.2012.53

[23] Okazaki T, Maeda A, Nishimura H, Kurosaki T, Honjo T. PD-1 immunoreceptor inhibits B cell receptor-mediated signaling by recruiting src homology 2-domain-containing tyrosine phosphatase 2 to phosphotyrosine. Proc Natl Acad Sci U S A. 2001;98(24):13866–71. 11698646 10.1073/pnas.231486598 PMC61133

[24] Sheppard KA, Fitz LJ, Lee JM, Benander C, George JA, Wooters J, PD-1 inhibits T-cell receptor induced phosphorylation of the ZAP70/CD3zeta signalosome and downstream signaling to PKCtheta. FEBS Lett. 2004;574(1–3):37–41. 15358536 10.1016/j.febslet.2004.07.083

[25] Chemnitz JM, Parry RV, Nichols KE, June CH, Riley JL. SHP-1 and SHP-2 associate with immunoreceptor tyrosine-based switch motif of programmed death 1 upon primary human T cell stimulation, but only receptor ligation prevents T cell activation. J Immunol. 2004;173(2):945–54. 15240681 10.4049/jimmunol.173.2.945

[26] Parry RV, Chemnitz JM, Frauwirth KA, Lanfranco AR, Braunstein I, Kobayashi SV, CTLA-4 and PD-1 receptors inhibit T-cell activation by distinct mechanisms. Mol Cell Biol. 2005;25(21):9543–53. 16227604 10.1128/MCB.25.21.9543-9553.2005 PMC1265804

[27] Riley JL. PD-1 signaling in primary T cells. Immunol Rev. 2009;229(1):114–25. 19426218 10.1111/j.1600-065X.2009.00767.x PMC3424066

[28] Fife BT, Pauken KE, Eagar TN, Obu T, Wu J, Tang Q, Interactions between PD-1 and PD-L1 promote tolerance by blocking the TCR-induced stop signal. Nat Immunol. 2009;10(11):1185–92. 19783989 10.1038/ni.1790 PMC2778301

[29] Karwacz K, Bricogne C, MacDonald D, Arce F, Bennett CL, Collins M, PD-L1 co-stimulation contributes to ligand-induced T cell receptor down-modulation on CD8^+^ T cells. EMBO Mol Med. 2011;3(10):581–92. 21739608 10.1002/emmm.201100165 PMC3191120

[30] Yokosuka T, Takamatsu M, Kobayashi-Imanishi W, Hashimoto-Tane A, Azuma M, Saito T. Programmed cell death 1 forms negative costimulatory microclusters that directly inhibit T cell receptor signaling by recruiting phosphatase SHP2. J Exp Med. 2012;209(6):1201–17. 22641383 10.1084/jem.20112741 PMC3371732

[31] Patsoukis N, Brown J, Petkova V, Liu F, Li L, Boussiotis VA. Selective effects of PD-1 on Akt and Ras pathways regulate molecular components of the cell cycle and inhibit T cell proliferation. Sci Signal. 2012;5(230):ra46. 22740686 10.1126/scisignal.2002796 PMC5498435

[32] Patsoukis N, Li L, Sari D, Petkova V, Boussiotis VA. PD-1 increases PTEN phosphatase activity while decreasing PTEN protein stability by inhibiting casein kinase 2. Mol Cell Biol. 2013;33(16):3091–8. 23732914 10.1128/MCB.00319-13 PMC3753920

[33] Hui E, Cheung J, Zhu J, Su X, Taylor MJ, Wallweber HA, T cell costimulatory receptor CD28 is a primary target for PD-1-mediated inhibition. Science. 2017;355(6332):1428–33. 28280247 10.1126/science.aaf1292 PMC6286077

[34] Mizuno R, Sugiura D, Shimizu K, Maruhashi T, Watada M, Okazaki IM, PD-1 primarily targets TCR signal in the inhibition of functional T cell activation. Front Immunol. 2019;10:630. 31001256 10.3389/fimmu.2019.00630 PMC6455061

[35] Celis-Gutierrez J, Blattmann P, Zhai Y, Jarmuzynski N, Ruminski K, Gregoire C, Quantitative interactomics in primary T cells provides a rationale for concomitant PD-1 and BTLA coinhibitor blockade in cancer immunotherapy. Cell Rep. 2019;27(11):3315–30.e7. 31189114 10.1016/j.celrep.2019.05.041 PMC6581740

[36] Patsoukis N, Duke-Cohan JS, Chaudhri A, Aksoylar HI, Wang Q, Council A, Interaction of SHP-2 SH2 domains with PD-1 ITSM induces PD-1 dimerization and SHP-2 activation. Commun Biol. 2020;3(1):128. 32184441 10.1038/s42003-020-0845-0 PMC7078208

[37] Regulation Chi H. and function of mTOR signalling in T cell fate decisions. Nat Rev Immunol. 2012;12(5):325–38. 22517423 10.1038/nri3198 PMC3417069

[38] Siska PJ, Rathmell JC. T cell metabolic fitness in antitumor immunity. Trends Immunol. 2015;36(4):257–64. 25773310 10.1016/j.it.2015.02.007 PMC4393792

[39] Patsoukis N, Bardhan K, Chatterjee P, Sari D, Liu B, Bell LN, PD-1 alters T-cell metabolic reprogramming by inhibiting glycolysis and promoting lipolysis and fatty acid oxidation. Nat Commun. 2015;6:6692. 25809635 10.1038/ncomms7692 PMC4389235

[40] Calder PC. The relationship between the fatty acid composition of immune cells and their function. Prostaglandins Leukot Essent Fatty Acids. 2008;79(3–5):101–8. 18951005 10.1016/j.plefa.2008.09.016

[41] Nicolaou A, Mauro C, Urquhart P, Marelli-Berg F. Polyunsaturated Fatty Acid-derived lipid mediators and T cell function. Front Immunol. 2014;5:75. 24611066 10.3389/fimmu.2014.00075 PMC3933826

[42] Xu S, Chaudhary O, Rodriguez-Morales P, Sun X, Chen D, Zappasodi R, Uptake of oxidized lipids by the scavenger receptor CD36 promotes lipid peroxidation and dysfunction in CD8(+) T cells in tumors. Immunity. 2021;54(7):1561–77.e7. 34102100 10.1016/j.immuni.2021.05.003 PMC9273026

[43] Tkachev V, Goodell S, Opipari AW, Hao LY, Franchi L, Glick GD, Programmed death-1 controls T cell survival by regulating oxidative metabolism. J Immunol. 2015;194(12):5789–800. 25972478 10.4049/jimmunol.1402180 PMC4562423

[44] Chamoto K, Chowdhury PS, Kumar A, Sonomura K, Matsuda F, Fagarasan S, Mitochondrial activation chemicals synergize with surface receptor PD-1 blockade for T cell-dependent antitumor activity. Proc Natl Acad Sci U S A. 2017;114(5):E761–70. 28096382 10.1073/pnas.1620433114 PMC5293087

[45] Priyadharshini B, Loschi M, Newton RH, Zhang JW, Finn KK, Gerriets VA, Cutting Edge: TGF-beta and phosphatidylinositol 3-kinase signals modulate distinct metabolism of regulatory T cell subsets. J Immunol. 2018;201(8):2215–9. 30209190 10.4049/jimmunol.1800311 PMC6179917

[46] Francisco LM, Salinas VH, Brown KE, Vanguri VK, Freeman GJ, Kuchroo VK, PD-L1 regulates the development, maintenance, and function of induced regulatory T cells. J Exp Med. 2009;206(13):3015–29. 20008522 10.1084/jem.20090847 PMC2806460

[47] Mariathasan S, Turley SJ, Nickles D, Castiglioni A, Yuen K, Wang Y, TGFbeta attenuates tumour response to PD-L1 blockade by contributing to exclusion of T cells. Nature. 2018;554(7693):544–8. 29443960 10.1038/nature25501 PMC6028240

[48] Lan Y, Zhang D, Xu C, Hance KW, Marelli B, Qi J, Enhanced preclinical antitumor activity of M7824, a bifunctional fusion protein simultaneously targeting PD-L1 and TGF-beta. Sci Transl Med. 2018;10(424):eaan5488. 29343622 10.1126/scitranslmed.aan5488

[49] Ravi R, Noonan KA, Pham V, Bedi R, Zhavoronkov A, Ozerov IV, Bifunctional immune checkpoint-targeted antibody-ligand traps that simultaneously disable TGFbeta enhance the efficacy of cancer immunotherapy. Nat Commun. 2018;9(1):741. 29467463 10.1038/s41467-017-02696-6 PMC5821872

[50] Gulley JL, Schlom J, Barcellos-Hoff MH, Wang XJ, Seoane J, Audhuy F, Dual inhibition of TGF-beta and PD-L1: a novel approach to cancer treatment. Mol Oncol. 2021 Dec 1. doi: 10.1002/1878-0261 PMC9168966 34854206

[51] Lan Y, Moustafa M, Knoll M, Xu C, Furkel J, Lazorchak A, Simultaneous targeting of TGF-beta/PD-L1 synergizes with radiotherapy by reprogramming the tumor microenvironment to overcome immune evasion. Cancer Cell. 2021;39(10):1388–403.e10. 34506739 10.1016/j.ccell.2021.08.008

[52] Quigley M, Pereyra F, Nilsson B, Porichis F, Fonseca C, Eichbaum Q, Transcriptional analysis of HIV-specific CD8^+^ T cells shows that PD-1 inhibits T cell function by upregulating BATF. Nat Med. 2010;16(10):1147–51. 20890291 10.1038/nm.2232 PMC3326577

[53] Xu C, Fu Y, Liu S, Trittipo J, Lu X, Qi R, BATF regulates T regulatory cell functional specification and fitness of triglyceride metabolism in restraining allergic responses. J Immunol. 2021;206(9):2088–100. 33879580 10.4049/jimmunol.2001184 PMC8442614

[54] Chen Y, Zander RA, Wu X, Schauder DM, Kasmani MY, Shen J, BATF regulates progenitor to cytolytic effector CD8(+) T cell transition during chronic viral infection. Nat Immunol. 2021;22(8):996–1007. 34282329 10.1038/s41590-021-00965-7 PMC9258987

[55] Seo H, Gonzalez-Avalos E, Zhang W, Ramchandani P, Yang C, Lio CJ, BATF and IRF4 cooperate to counter exhaustion in tumor-infiltrating CAR T cells. Nat Immunol. 2021;22(8):983–95. 34282330 10.1038/s41590-021-00964-8 PMC8319109

[56] Chikuma S, Terawaki S, Hayashi T, Nabeshima R, Yoshida T, Shibayama S, PD-1-mediated suppression of IL-2 production induces CD8^+^ T cell anergy in vivo. J Immunol. 2009;182(11):6682–9. 19454662 10.4049/jimmunol.0900080

[57] Charlton JJ, Chatzidakis I, Tsoukatou D, Boumpas DT, Garinis GA, Mamalaki C. Programmed death-1 shapes memory phenotype CD8 T cell subsets in a cell-intrinsic manner. J Immunol. 2013;190(12):6104–14. 23686498 10.4049/jimmunol.1201617

[58] Galletti G, De Simone G, Mazza EMC, Puccio S, Mezzanotte C, Bi TM, Two subsets of stem-like CD8(+) memory T cell progenitors with distinct fate commitments in humans. Nat Immunol. 2020;21(12):1552–62. 33046887 10.1038/s41590-020-0791-5 PMC7610790

[59] Johnnidis JB, Muroyama Y, Ngiow SF, Chen Z, Manne S, Cai Z, Inhibitory signaling sustains a distinct early memory CD8(+) T cell precursor that is resistant to DNA damage. Sci Immunol. 2021;6(55):eabe3702. 33452106 10.1126/sciimmunol.abe3702 PMC8258400

[60] Kalia V, Yuzefpolskiy Y, Vegaraju A, Xiao H, Baumann F, Jatav S, Metabolic regulation by PD-1 signaling promotes long-lived quiescent CD8 T cell memory in mice. Sci Transl Med. 2021;13(615):eaba6006. 34644150 10.1126/scitranslmed.aba6006 PMC8896520

[61] Park HJ, Kusnadi A, Lee EJ, Kim WW, Cho BC, Lee IJ, Tumor-infiltrating regulatory T cells delineated by upregulation of PD-1 and inhibitory receptors. Cell Immunol. 2012;278(1–2):76–83. 23121978 10.1016/j.cellimm.2012.07.001

[62] Kamada T, Togashi Y, Tay C, Ha D, Sasaki A, Nakamura Y, PD-1(+) regulatory T cells amplified by PD-1 blockade promote hyperprogression of cancer. Proc Natl Acad Sci U S A. 2019;116(20):9999–10008. 31028147 10.1073/pnas.1822001116 PMC6525547

[63] Kumagai S, Togashi Y, Kamada T, Sugiyama E, Nishinakamura H, Takeuchi Y, The PD-1 expression balance between effector and regulatory T cells predicts the clinical efficacy of PD-1 blockade therapies. Nat Immunol. 2020;21(11):1346–58. 32868929 10.1038/s41590-020-0769-3

[64] Kumagai S, Koyama S, Itahashi K, Tanegashima T, Lin Y-T, Togashi Y, Lactic acid promotes PD-1 expression in regulatory T cells in highly glycolytic tumor microenvironments. Cancer Cell. 2022;40:1–18. 35090594 10.1016/j.ccell.2022.01.001

[65] Tan CL, Kuchroo JR, Sage PT, Liang D, Francisco LM, Buck J, PD-1 restraint of regulatory T cell suppressive activity is critical for immune tolerance. J Exp Med. 2021;218(1):e20182232. 33045061 10.1084/jem.20182232 PMC7543091

[66] Michalek RD, Gerriets VA, Jacobs SR, Macintyre AN, Maciver NJ, Mason EF, Cutting Edge: Distinct Glycolytic and Lipid Oxidative Metabolic Programs Are Essential for Effector and Regulatory CD4^+^ T Cell Subsets. J Immunol. 2011;186(6):3299–303. 21317389 10.4049/jimmunol.1003613 PMC3198034

[67] Crellin NK, Garcia RV, Levings MK. Altered activation of AKT is required for the suppressive function of human CD4^+^CD25^+^ T regulatory cells. Blood. 2007;109(5):2014–22. 17062729 10.1182/blood-2006-07-035279

[68] Haxhinasto S, Mathis D, Benoist C. The AKT-mTOR axis regulates de novo differentiation of CD4^+^ Foxp3^+^ cells. J Exp Med. 2008;205:565–74. 18283119 10.1084/jem.20071477 PMC2275380

[69] Huang X, Venet F, Wang YL, Lepape A, Yuan Z, Chen Y, PD-1 expression by macrophages plays a pathologic role in altering microbial clearance and the innate inflammatory response to sepsis. Proc Natl Acad Sci U S A. 2009;106(15):6303–8. 19332785 10.1073/pnas.0809422106 PMC2669369

[70] Chen W, Wang J, Jia L, Liu J, Tian Y. Attenuation of the programmed cell death-1 pathway increases the M1 polarization of macrophages induced by zymosan. Cell Death Dis. 2016;7:e2115. 26913605 10.1038/cddis.2016.33 PMC4849159

[71] Shen L, Gao Y, Liu Y, Zhang B, Liu Q, Wu J, PD-1/PD-L pathway inhibits M.tb-specific CD4(+) T-cell functions and phagocytosis of macrophages in active tuberculosis. Sci Rep. 2016;6:38362. 27924827 10.1038/srep38362 PMC5141449

[72] Gordon SR, Maute RL, Dulken BW, Hutter G, George BM, McCracken MN, PD-1 expression by tumour-associated macrophages inhibits phagocytosis and tumour immunity. Nature. 2017;545(7655):495–9. 28514441 10.1038/nature22396 PMC5931375

[73] Zhang Y, Zhou Y, Lou J, Li J, Bo L, Zhu K, PD-L1 blockade improves survival in experimental sepsis by inhibiting lymphocyte apoptosis and reversing monocyte dysfunction. Crit Care. 2010;14(6):R220. 21118528 10.1186/cc9354 PMC3220038

[74] Qorraj M, Bruns H, Bottcher M, Weigand L, Saul D, Mackensen A, The PD-1/PD-L1 axis contributes to immune metabolic dysfunctions of monocytes in chronic lymphocytic leukemia. Leukemia. 2017;31(2):470–8. 27479178 10.1038/leu.2016.214

[75] Strauss L, Mahmoud MAA, Weaver JD, Tijaro-Ovalle NM, Christofides A, Wang Q, Targeted deletion of PD-1 in myeloid cells induces antitumor immunity. Sci Immunol. 2020;5(43):eaay1863. 31901074 10.1126/sciimmunol.aay1863 PMC7183328

[76] Yu Y, Tsang JC, Wang C, Clare S, Wang J, Chen X, Single-cell RNA-seq identifies a PD-1(hi) ILC progenitor and defines its development pathway. Nature. 2016;539(7627):102–6. 27749818 10.1038/nature20105

[77] Boussiotis VA. Molecular and biochemical aspects of the PD-1 checkpoint pathway. N Engl J Med. 2016;375(18):1767–78. 27806234 10.1056/NEJMra1514296 PMC5575761

[78] Nakamura K, Smyth MJ. Myeloid immunosuppression and immune checkpoints in the tumor microenvironment. Cell Mol Immunol. 2020;17(1):1–12. 31611651 10.1038/s41423-019-0306-1 PMC6952382

[79] Quatrini L, Mariotti FR, Munari E, Tumino N, Vacca P, Moretta L. The immune checkpoint PD-1 in natural killer cells: Expression, function and targeting in tumour immunotherapy. Cancers. 2020;12(11):3285. 10.3390/cancers12113285 PMC7694632 33172030

[80] Westerterp M, Gautier EL, Ganda A, Molusky MM, Wang W, Fotakis P, Cholesterol accumulation in dendritic cells links the inflammasome to acquired immunity. Cell Metab. 2017;25(6):1294–304.e6. 28479366 10.1016/j.cmet.2017.04.005 PMC5514787

[81] Vivier E, Raulet DH, Moretta A, Caligiuri MA, Zitvogel L, Lanier LL, Innate or adaptive immunity? The example of natural killer cells. Science. 2011;331(6013):44–9. 21212348 10.1126/science.1198687 PMC3089969

[82] Vivier E, van de Pavert SA, Cooper MD, Belz GT. The evolution of innate lymphoid cells. Nat Immunol. 2016;17(7):790–4. 27328009 10.1038/ni.3459 PMC5287353

[83] Donnelly RP, Loftus RM, Keating SE, Liou KT, Biron CA, Gardiner CM, mTORC1-dependent metabolic reprogramming is a prerequisite for NK cell effector function. J Immunol. 2014;193(9):4477–84. 25261477 10.4049/jimmunol.1401558 PMC4201970

[84] Ali AK, Nandagopal N, Lee SH. IL-15-PI3K-AKT-mTOR: A critical pathway in the life journey of natural killer cells. Front Immunol. 2015;6:355. 26257729 10.3389/fimmu.2015.00355 PMC4507451

[85] Beldi-Ferchiou A, Lambert M, Dogniaux S, Vely F, Vivier E, Olive D, PD-1 mediates functional exhaustion of activated NK cells in patients with Kaposi sarcoma. Oncotarget. 2016;7(45):72961–77. 27662664 10.18632/oncotarget.12150 PMC5341956

[86] Liu Y, Cheng Y, Xu Y, Wang Z, Du X, Li C, Increased expression of programmed cell death protein 1 on NK cells inhibits NK-cell-mediated anti-tumor function and indicates poor prognosis in digestive cancers. Oncogene. 2017;36(44):6143–53. 28692048 10.1038/onc.2017.209 PMC5671935

[87] Pesce S, Greppi M, Tabellini G, Rampinelli F, Parolini S, Olive D, Identification of a subset of human natural killer cells expressing high levels of programmed death 1: A phenotypic and functional characterization. J Allergy Clin Immunol. 2017;139(1):335–46.e3. 27372564 10.1016/j.jaci.2016.04.025

[88] Hsu J, Hodgins JJ, Marathe M, Nicolai CJ, Bourgeois-Daigneault MC, Trevino TN, Contribution of NK cells to immunotherapy mediated by PD-1/PD-L1 blockade. J Clin Invest. 2018;128(10):4654–68. 30198904 10.1172/JCI99317 PMC6159991

[89] Quatrini L, Wieduwild E, Escaliere B, Filtjens J, Chasson L, Laprie C, Endogenous glucocorticoids control host resistance to viral infection through the tissue-specific regulation of PD-1 expression on NK cells. Nat Immunol. 2018;19(9):954–62. 30127438 10.1038/s41590-018-0185-0 PMC6138242

[90] Quatrini L, Vacca P, Tumino N, Besi F, Di Pace AL, Scordamaglia F, Glucocorticoids and the cytokines IL-12, IL-15, and IL-18 present in the tumor microenvironment induce PD-1 expression on human natural killer cells. J Allergy Clin Immunol. 2021;147(1):349–60. 32417134 10.1016/j.jaci.2020.04.044

[91] Ansari MJ, Salama AD, Chitnis T, Smith RN, Yagita H, Akiba H, The programmed death-1 (PD-1) pathway regulates autoimmune diabetes in nonobese diabetic (NOD) mice. J Exp Med. 2003;198(1):63–9. 12847137 10.1084/jem.20022125 PMC2196083

[92] Paterson AM, Brown KE, Keir ME, Vanguri VK, Riella LV, Chandraker A, The programmed death-1 ligand 1:B7–1 pathway restrains diabetogenic effector T cells in vivo. J Immunol. 2011;187:1097–105. 21697456 10.4049/jimmunol.1003496 PMC3148082

[93] Bu DX, Tarrio M, Maganto-Garcia E, Stavrakis G, Tajima G, Lederer J, Impairment of the programmed cell death-1 pathway increases atherosclerotic lesion development and inflammation. Arterioscler Thromb Vasc Biol. 2011;31(5):1100–7. 21393583 10.1161/ATVBAHA.111.224709 PMC3104026

[94] Yang W, Bai Y, Xiong Y, Zhang J, Chen S, Zheng X, Potentiating the antitumour response of CD8(+) T cells by modulating cholesterol metabolism. Nature. 2016;531(7596):651–5. 26982734 10.1038/nature17412 PMC4851431

[95] Drobni ZD, Alvi RM, Taron J, Zafar A, Murphy SP, Rambarat PK, Association between immune checkpoint inhibitors with cardiovascular events and atherosclerotic plaque. Circulation. 2020;142(24):2299–311. 33003973 10.1161/CIRCULATIONAHA.120.049981 PMC7736526

[96] Miyajima M, Zhang B, Sugiura Y, Sonomura K, Guerrini MM, Tsutsui Y, Metabolic shift induced by systemic activation of T cells in PD-1-deficient mice perturbs brain monoamines and emotional behavior. Nat Immunol. 2017;18(12):1342–52. 29058703 10.1038/ni.3867

[97] Li H, Bullock K, Gurjao C, Braun D, Shukla SA, Bosse D, Metabolomic adaptations and correlates of survival to immune checkpoint blockade. Nat Commun. 2019;10(1):4346. 31554815 10.1038/s41467-019-12361-9 PMC6761178

[98] Hatae R, Chamoto K, Kim YH, Sonomura K, Taneishi K, Kawaguchi S, Combination of host immune metabolic biomarkers for the PD-1 blockade cancer immunotherapy. JCI Insight. 2020;5(2):e133501. 10.1172/jci.insight.133501 PMC7098729 31855576

[99] Arguello RJ, Combes AJ, Char R, Gigan JP, Baaziz AI, Bousiquot E, SCENITH: A Flow Cytometry-Based Method to Functionally Profile Energy Metabolism with Single-Cell Resolution. Cell Metab. 2020;32(6):1063–75.e7. 33264598 10.1016/j.cmet.2020.11.007 PMC8407169

